# A Novel Type of PD-L1 Inhibitor rU1 snRNPA From Human-Derived Protein Scaffolds Library

**DOI:** 10.3389/fonc.2021.781046

**Published:** 2021-11-29

**Authors:** Chuang Ma, Sennan Qiao, Zhiyi Liu, Liang Shan, Chongyang Liang, Meiling Fan, Fei Sun

**Affiliations:** ^1^ School of Pharmaceutical Sciences, Jilin University, Changchun, China; ^2^ Institute of Frontier Medical Science, Jilin University, Changchun, China; ^3^ Jilin Academy of Chinese Medicine Sciences, Changchun, China

**Keywords:** PD-L1, inhibitor, human-derived, scaffold, melanoma, breast cancer

## Abstract

Three marketed anti-PD-L1 antibodies almost have severe immune-mediated side effects. The therapeutic effects of anti-PD-L1 chemical inhibitors are not satisfied in the clinical trials. Here we constructed human-derived protein scaffolds library and screened scaffolds with a shape complementary to the PD-1 binding domain of PD-L1. The RNA binding domain of U1 snRNPA was selected as one of potential binders because it had the most favorable binding energies with PD-L1 and conformed to pre-established biological criteria for the screening of candidates. The recombinant U1 snRNPA (rU1 snRNPA) in *Escherichia coli* exhibits anti-cancer activity in melanoma and breast cancer by reactivating tumor-suppressed T cells *in vitro* and anti-melanoma activity *in vivo*. Considering hydrophobic and electrostatic interactions, three residues were mutated on the interface of U1 snRNPA and PD-L1 complex, and the ranked variants by PatchDock and A32D showed an increased active phenotype. The screening of human-derived protein scaffolds may become the potential development of therapeutic agents.

## Introduction

Recently, immune checkpoint inhibitors have developed rapidly and become the most promising cancer immunotherapy strategy with notable clinical benefits (1). The PD-1/PD-L1 axis is one of the most typical immune checkpoint axes, and its inhibitors were acknowledged as the fourth major cancer therapy (2). Emerging evidence shows that the use of antibodies to block the interaction between PD-L1 and its receptors can strengthen the cytotoxic activity of anti-tumor T cells and alleviate PD-L1-dependent immunosuppressive effects *in vitro* (3). Six monoclonal antibodies (mAbs) have been approved by the Food and Drug Administration (FDA) for use in cancer immunotherapy, including durvalumab, cemiplimab, nivolumab, pembrolizumab, avelumab, and atezolizumab (4). However, antibodies have intrinsic disadvantages that limit their application—for example, high manufacturing costs, low instability, low tissue penetration, and immunogenicity (5). Therefore, drug discovery studies relating to the PD-1/PD-L1 axis have increasingly focused on low-molecular-weight inhibitors such as single-chain antibodies, chemical inhibitors, peptides, and peptidomimetics (6). However, the binding interface of PD-1 and PD-L1 is large and flat and lacks deep pockets; some chemical inhibitors are prone to off-targeting (7). The current development of chemical inhibitors is focused on inducing PD-L1 dimerization rather than directly blocking, but so far, only publications and patents of PD-1/PD-L1 chemical inhibitors have been disclosed; there are no FDA-approved inhibitors for clinical use, and some chemical inhibitors failed to reactivate T cells and were cytotoxic (8). Therefore, it is still an important proposition to find the direction of a novel molecular structure for therapeutic use. Because immunogenicity is an important issue to develop therapeutic agents, soluble human-derived protein scaffolds are an ideal research and development direction.

However, due to the limitations in computational resources and other aspects, it is still very difficult to predict the interactions between macromolecules in batches. Molecular docking is a computer-aided drug design method based on receptors, starting from ligand–receptor binding, and theoretically calculating and analyzing the interaction modes between ligand and receptor (9). Molecular docking in drug screening mainly focuses on virtual screening and activation prediction with small molecules as ligands (10), while it is less used in drug screening with protein ligands (11). PatchDock uses object recognition and image segmentation techniques similar to those used in computer vision. The surface of a given molecule can be divided into multiple small patches according to the shape by PatchDock. Once the complementary structure is identified, it can be superimposed using a shape-matching algorithm and finally ranked by shape complementarity score (12). Protein scaffolds originally represent a category of affinity proteins that complement the immunoglobulins and antibody derivatives (13). Non-immunoglobulin-based protein scaffolds have been reported as promising alternatives to traditional monoclonal antibodies in recent years (13). The idea of using protein scaffolds as PD-L1 inhibitors originally came from the basic mechanism through which antibodies are produced against antigens. The amino acids in CDRs act as protein scaffolds which can produce diverse structures and form the complementary shape to recognize specific epitopes (14). The process of ligands or inhibitors binding to target proteins is similar to the binding of antigen and antibody, so protein scaffolds, especially human-derived protein scaffolds like the amino acids in complementarity-determining regions, whose shape is complementary to the target protein, have a significant potential to be ideal inhibitors.

**Scheme 1:**
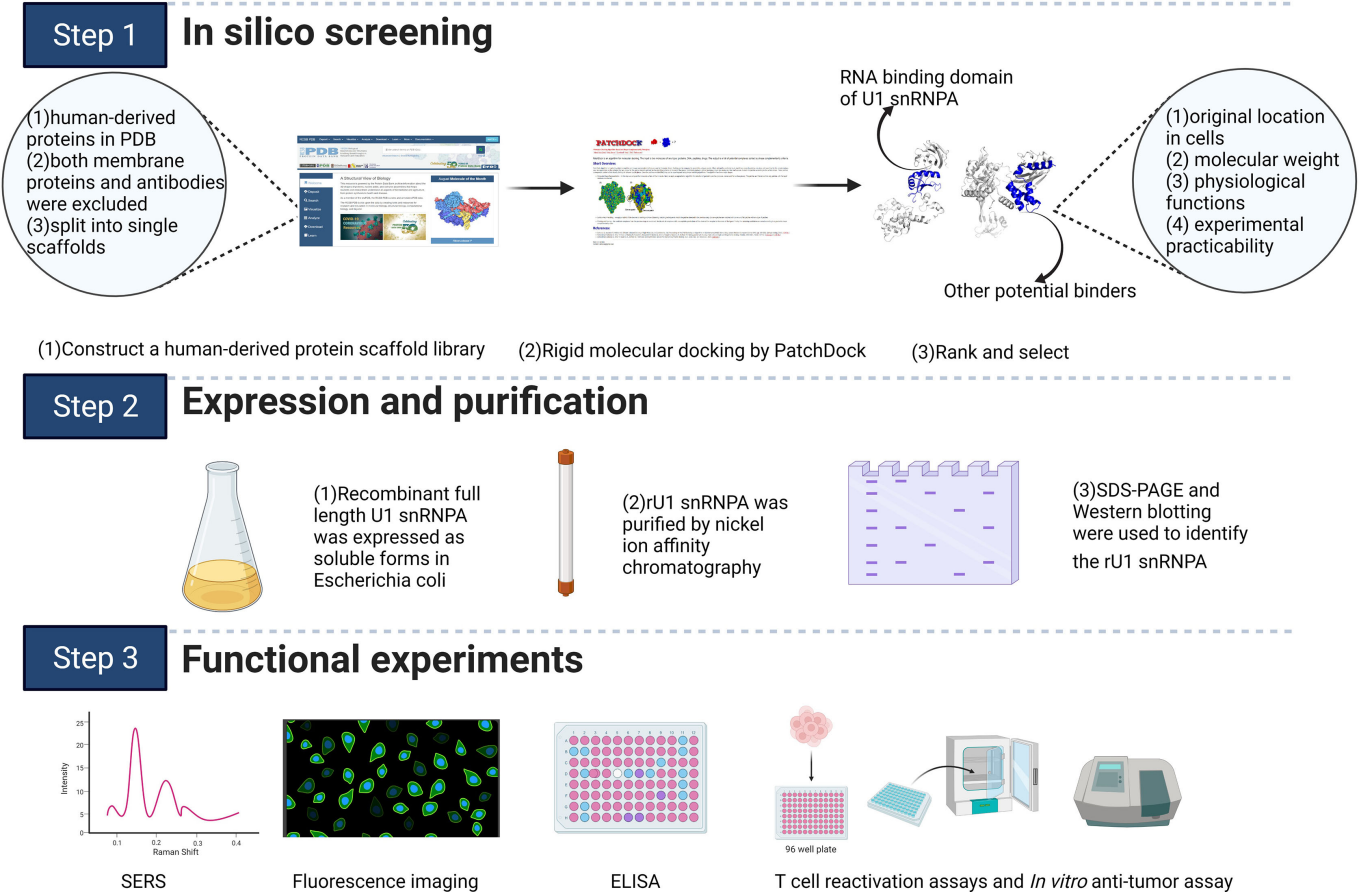
Schematic illustration of the study. The scheme was created with BioRender.com.

In this study, we used rigid molecular docking server PatchDock to screen PD-L1 inhibitors from a human-derived protein scaffolds library (**Scheme 1**). The RNA binding domain of U1 snRNPA was selected as a protein binder to the PD-1 binding domain of PD-L1. Recombinant full-length U1 snRNPA in *Escherichia coli* was proven to inhibit PD-1/PD-L1 interaction directly. The results were demonstrated by T cell reactivation assay and anti-cancer efficacy assay *in vitro*/*in vivo*. The rU1 snRNPA is considered a lead compound; we further mutated its residues on the interface of U1 snRNPA and PD-L1 complex, and the variant A32D showed an active phenotype. Our results suggested that the application of a human-derived protein scaffolds library and rigid molecular docking is a highly efficient and rapid tool for designing novel therapeutic protein drugs.

## Materials and Methods

### Establishment of a Human-Derived Protein Scaffolds Library

A human-derived protein scaffolds library was established by the following three steps. First, all human-derived proteins in the RCSB Protein Data Bank (PDB; https://www.rcsb.org/) (15) were selected using the category “Organisms: *Homo sapiens*”. Second, both membrane proteins and antibodies were excluded using the annotation “transmembrane proteins” and searching for the keyword “antibody” in the PDB, respectively (16). Third, the selected structures were split into single scaffolds using “END” in the PDB file. Considering the available computing ability and the huge number of samples, 1,863 scaffolds were randomly selected and used as a library for screening.

### Screening of PD-L1 Binding Scaffolds by Rigid Molecular Docking

The PD-1 binding domain from the human PD-L1 structure (PDB: 5C3T) and the constructed library were defined as the corresponding docking analytes. PatchDock was chosen as the molecular docking program (http://bioinfo3d.cs.tau.ac.il/PatchDock/) (17). PatchDock is a geometry-based molecular docking algorithm. The algorithm has three major stages: molecular shape representation, surface patch matching, and filtering and scoring. The parameters in PatchDock were all set to default values. The PatchDock score of PD-L1 and durvalumab, which has a proven high affinity for PD-L1, was set as a standard value. Only scaffolds with scores close to or higher than this standard value were selected in the first round of screening. For the second-round screening, the top 10 docking models for each scaffold–PD-L1 docking result according to the PatchDock score were downloaded, followed by analysis and assessment of their binding modes and conformations. Scaffolds with similar paratopes to PD-1/PD-L1 were selected (18). The geometric shape complementarity scores of these scaffolds were recorded and ranked. In the third round, the scaffolds were screened further based on their original location, molecular weight, physiological function, and experimental practicability in the laboratory. The RNA-binding domain of the U1 small nuclear ribonucleoprotein A (PDB: 1U6B) was selected for the next step of the research. The key residues on the interaction surfaces of PD-L1 and U1 snRNPA were analyzed using InterProSurf (19).

### Inhibitory Effect on PD-1/PD-L1 Detected by SERS

A SERS-based PD-1/PD-L1 inhibitor detection platform was used to detect the inhibitory effect. When AgNPs@PD-1@4-ABP and MNs@PD-L1 bind normally, a high SERS characteristic peak would be produced; if the added substance can inhibit the binding between them, the characteristic peak would decrease. Therefore, the inhibitory effect on the PD-1/PD-L1 signaling pathway can be detected by observing whether the substance caused a reduction of the SERS characteristic peak. The feasibility of this method was preliminarily verified through experiments with existing inhibitors (durvalumab and BMS-202; see the supplementary materials for details).

### T Cell Reactivation Assays

Levels of IFN-γ or TNF-α production were evaluated to determine whether tumor-suppressed T cells were reactivated. Human CD4+ T lymphocytes were co-cultured with A375 cells, MDA-MB-231 cells, PD-L1-negative HEK293T cells, or HEK293T cells with hPD-L1 expression (HEK293T-hPD-L1) in 96-well plates. A density of 10,000 cells per well was plated and adhered for 24 h; then, 20,000 reactivated CD4+ T lymphocytes per well were added to the plates with different inhibitors, followed by co-culturing at 37°C, 5% CO_2_ for 48 h. The level of IFN-γ or TNF-α production in culture supernatants was detected by Human IFN-γ ELISA kit (BD Biosciences) and Human TNF-α ELISA kit (BD Biosciences) (for the expression and purification of rU1 snRNPA, binding ELISA, and competitive ELISA, *in vitro* anti-cancer assay, *in vivo* anti-melanoma assay, see the supplementary materials for details).

## Results

### U1 snRNPA Was Screened From Human-Derived Protein Scaffolds Library by Rigid Molecular Docking

**Figure 1:**
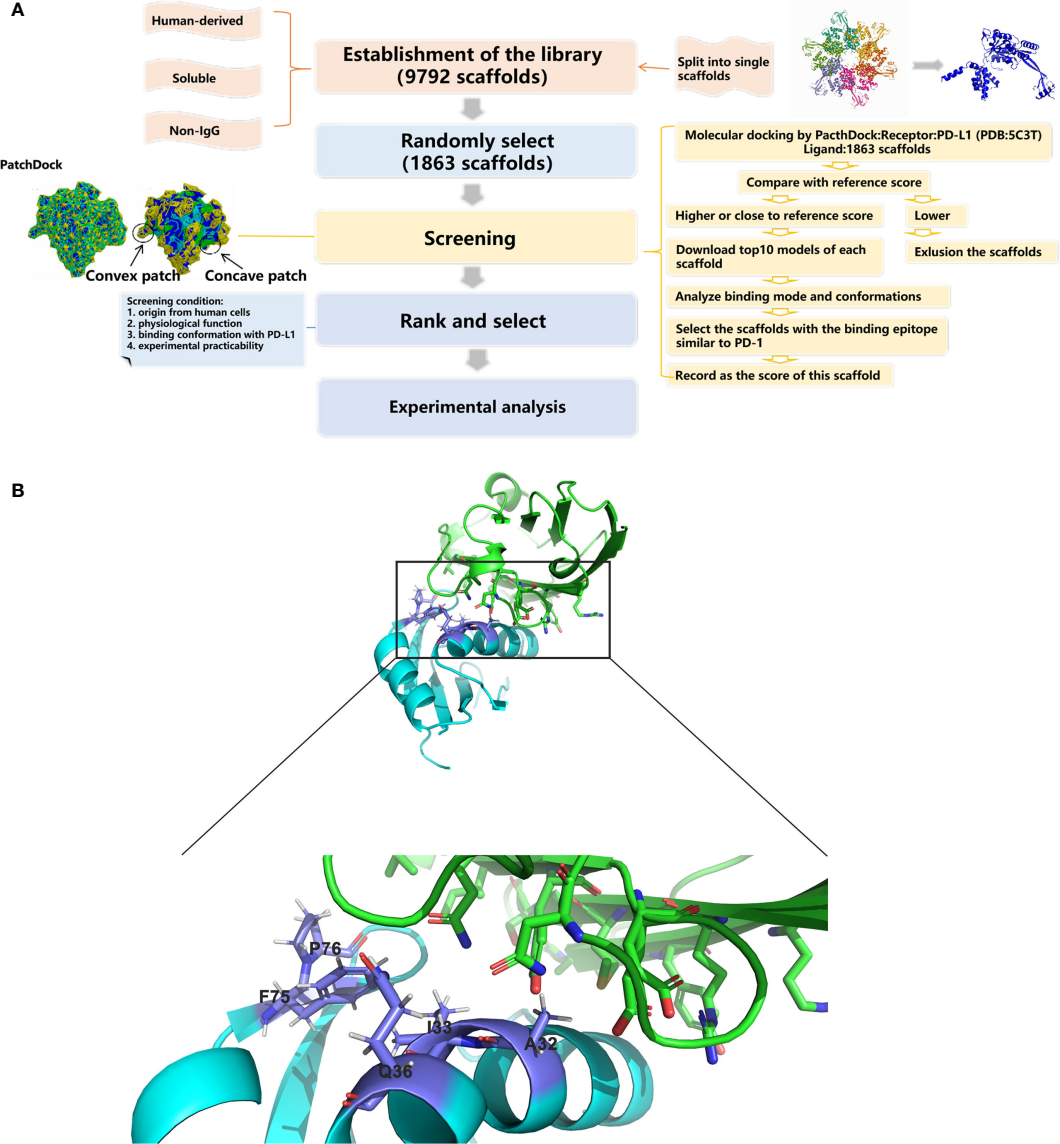
Schematic drawings of the screening processes and interactions of U1 snRNPA/PD-L1. **(A)** Flow chart of database construction and screening processes. **(B)** Cartoon representations for the identified interface of U1 snRNPA/PD-L1 complex. Cyan, U1 snRNPA (PDB: 1U6B); green, PD-L1. Stick representations show the key residues.

The whole screening process is illustrated in **Figure 1A**. A library containing 1,863 scaffolds of human-derived proteins was successfully constructed and screened for potential PD-L1 binding ability. The standard value for molecular docking was 16,172, which was the PatchDock score of PD-L1 and durvalumab. By two-round screening, the top 20 binders (**Supplementary Table S1**) were screened to exclude enzymes and their analogues. Finally, the remaining binders were screened based on their original location in the cells, molecular weight, physiological functions, and experimental practicability. The RNA-binding domain of the U1 small nuclear ribonucleoprotein A (U1 snRNPA) (PDB: 1U6B) was selected for the subsequent study.

The key residues of the interaction surfaces between U1 snRNPA and PD-L1 were analyzed by InterProSurf. As shown in **Figure 1B**, the α-helix of U1 snRNPA interacted with the loop of PD-L1. The binding sites were as follows: Ala32, Ile33, Gln36, Ser71, Phe75, and Pro76 in U1 snRNPA and Asp61, Arg113, and Tyr123 in PD-L1.

According to the *in silico* results, recombinant full-length U1 snRNPA was expressed as soluble forms in the *Escherichia coli* (E. coli) expression system. As the sequence of the RNA-binding domain of the U1 snRNPA (PDB: 1U6B) was too short to form the correct tertiary structure, the whole sequence of U1 snRNPA was synthesized and expressed for subsequent experiments, as it had the same binding epitope with PD-L1. After expression, rU1 snRNPA was purified successfully by nickel-ion-affinity chromatography (**Supplementary Figures S1A**, **S2**).

### rU1 snRNPA Inhibit PD-1/PD-L1 Interaction by Binding PD-L1

**Figure 2:**
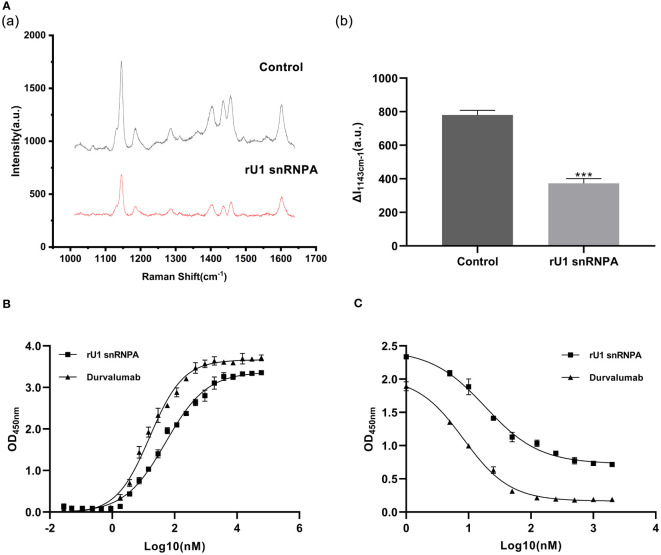
Results of binding ability to PD-L1 and inhibition effect on PD-1/PD-L1 interaction. **(A)** Comparison of SERS spectra of rU1 snRNPA (1.5 μM) and control. (a) Representative SERS spectra. *λ*_ex_ = 632.8 nm, *t* = 5 s, and accumulation times = 1. (b) Intensity of the characteristic peak of 4-amnobiphenyl with the results of three repeated experiments. Data are shown as mean ± SD. ***P < 0.001. **(B)** Binding curves of rU1 snRNPA and durvalumab. Curve fitting was performed by using GraphPad Prism 8.0. Error bars denote SD. The experiment was performed in double for each sample group. **(C)** Inhibition curves of rU1 snRNPA and durvalumab. Curve fitting was performed by using GraphPad Prism 8.0. Error bars denote SD. The experiment was performed in double for each sample group.

Based on the results of SERS, we have preliminarily verified the inhibition efficacy of rU1 snRNPA on PD-1/PD-L1 interaction in practice. The schematic illustration of the establishment of the SERS-based PD-1/PD-L1 inhibitor detection platform is shown in **Scheme S1**, and the results are shown in **Supplementary Figure S4**. The intensity of the characteristic peak of 4-amnobiphenyl (4-ABP) had significantly decreased compared to the control when rU1 snRNPA was added (**Figure 2A**), which indicated that rU1 snRNPA inhibited the interaction of AgNPs@PD-1@4-ABP and MNs@PD-L1. The results of ELISA further proved that rU1 snRNPA had the ideal binding ability to PD-L1 in competition with PD-1 (**Figures 2B, C**). The EC50 of rU1 snRNPA was 55.17 nM. The IC50 of rU1 snRNPA was 18.06 nM. Although the EC50 and IC50 of durvalumab (16.05 and 8.326 nM) were lower than those of rU1 snRNPA, these results still proved the enormous research potential of rU1 snRNPA as a primitive PD-L1 binder. SERS was used to analyze the protein–protein interaction in this study. Combined with the results of ELISA, it can be experimentally proved that rU1 snRNPA has the ability to inhibit the interaction of PD-1/PD-L1 by binding PD-L1, which further proved the effectiveness of the screening method in this study.

### rU1 snRNPA Reactivate Tumor-Suppressed T Cells and Had Anti-cancer Efficacy *In Vitro* and *In Vivo*

**Figure 3:**
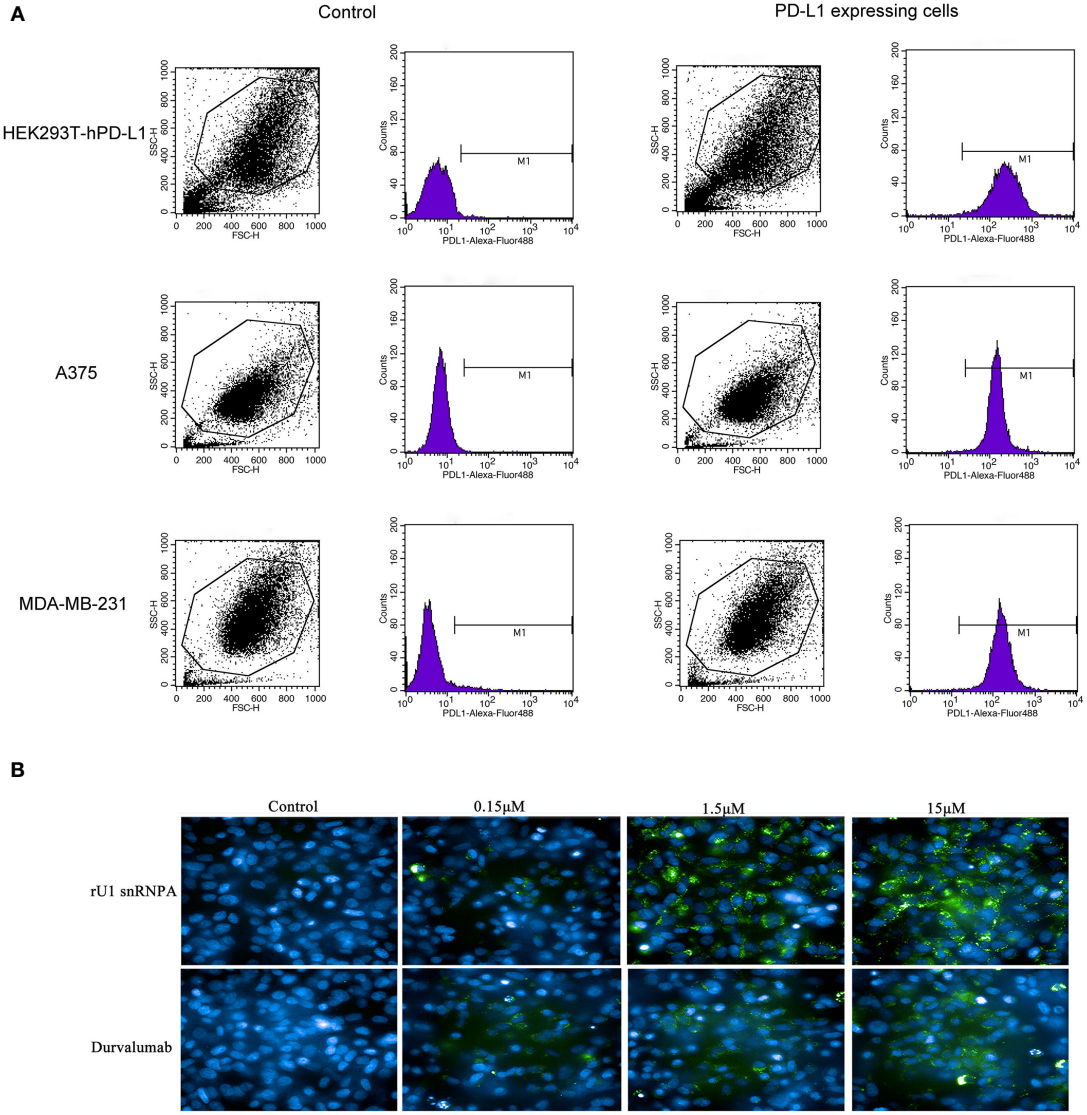
**(A)** Flow cytometry results of the expression of hPD-L1 on cells. **(B)** High content imaging of A375 cells with different concentrations of rU1 snRNPA or durvalumab. Blue, nucleus by Hoechst33342; green, rU1 snRNPA or durvalumab conjugated with Alexa Fluor^®^ 488 NHS Ester.

**Figure 4:**
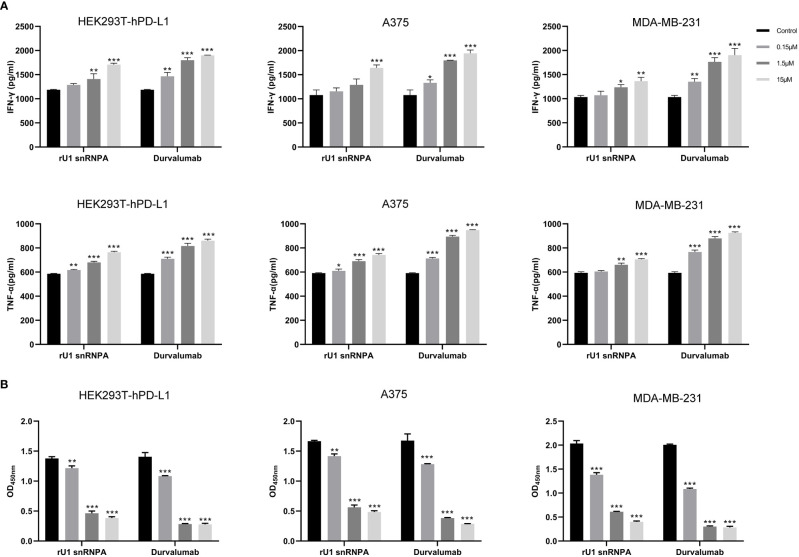
Effect of rU1 snRNPA on T cell reactivation function and anti-cancer efficacy. **(A)** Effect of rU1 snRNPA or durvalumab on IFN-γ and TNF-α secretion from CD4+ T cells co-cultured with HEK293T-hPD-L1 cells or A375 cells or MDA-MB-231 cells. **(B)** Anti-cancer efficacy of rU1 snRNPA or durvalumab mixed with CD4+ T cells in HEK293T-hPD-L1 cells or A375 cells or MDA-MB-231 cells. Data are shown as mean ± SD.*P < 0.05, **P < 0.01, ***P < 0.001.

PD-1/PD-L1 inhibitors work by reactivating tumor-suppressed T cells rather than directly killing tumor cells. In this study, the function of T cell reactivation of rU1 snRNPA was evaluated by measuring the secretion of cytokines IFN-γ and TNF-α in CD4+ T cells. In the T cell–tumor co-cultured assay, A375 cells, MDA-MB-231 cells, and HEK293T-hPD-L1 cells expressed human PD-L1, binding to PD-1 on CD4+ T cells to suppress the function of T cells. The verification of PD-L1 expression on cells by flow cytometry and high-content imaging of A375 cells is shown in **Figure 3**. When rU1 snRNPA was added, the levels of IFN-γ and TNF-α showed a dose-dependent increase similar with durvalumab (**Figure 4A**). Although some groups showed no significant differences at lower concentrations, the levels of IFN-γ and TNF-α were still higher than those of the control group. At the concentration of 15 μM, both IFN-γ and TNF-α secretion elevated significantly compared to the control group. PD-L1-negative HEK293T cells treated with rU1 snRNPA were used to confirm whether the observations were dependent on blocking the PD-1/PD-L1 interaction. The results showed that rU1 snRNPA did not significantly increase the IFN-γ and TNF-α secretion of PD-L1-negative HEK293T cells co-cultured with CD4+ T cells (**Supplementary Figure S5A**). These results indicated that rU1 snRNPA had T cell reactivation function by blocking the PD-1/PD-L1 interaction.

CCK-8 assay was used to evaluate the anti-melanoma and anti-breast cancer efficacy and killing activity of rU1 snRNPA in A375 cells, MDA-MB-231 cells, and HEK293T-hPD-L1 cells. After treatment with rU1 snRNPA or durvalumab mixed with CD4+ T cells, the cells were killed in a concentration-dependent manner, and all three concentrations showed a significant difference compared to the control group. Since 15 μM showed a little difference to 1.5 μM, the latter was considered as the ideal concentration for further development (**Figure 4B**). rU1 snRNPA showed little killing activity in A375 cells, MDA-MB-231 cells, or HEK293T-hPD-L1 cells without CD4+ T cells or PD-L1-negative HEK293T cells with CD4+ T cells (**Supplementary Figure S5B**), which proved that rU1 snRNPA only kills cells with CD4+ T cells and were not toxic to cells. rU1 snRNPA had *in vitro* anti-cancer efficacy.

**Figure 5:**
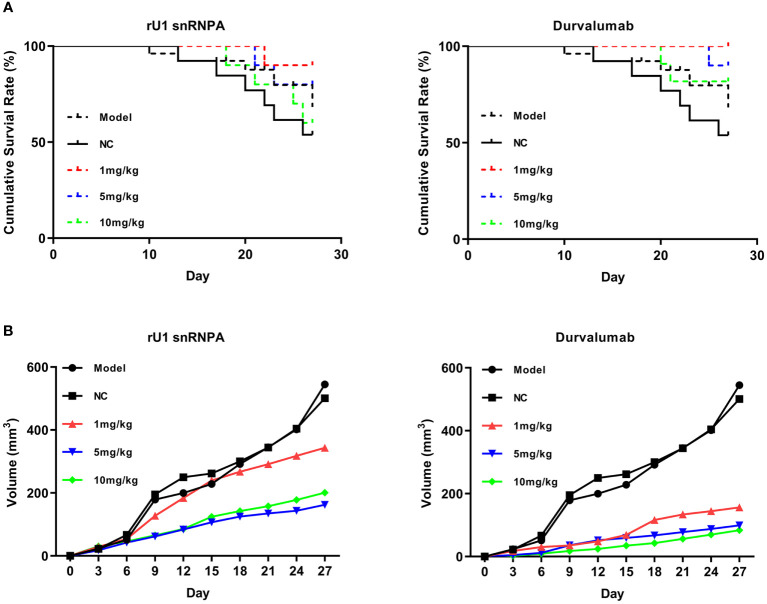
Anti-melanoma efficacy of rU1 snRNPA in a melanoma–human immune system immunodeficiency mouse model. **(A)** Survival rates of rU1 snRNPA or durvalumab. **(B)** Tumor volume changes measured over time in different groups (*n* = 10).

Given the promising results of *in vitro* anti-melanoma efficacy, a melanoma–human immune system immunodeficiency mouse model was used to further evaluate the *in vivo* anti-melanoma efficacy of rU1 snRNPA. In this mouse model, PBMCs supplied T cells, which reacted with melanoma A375 cells, and immune responses were reactivated to inhibit tumor growth. As shown in **Figure 5A**, rU1 snRNPA resulted in a similar survival period as that of durvalumab and both inhibited tumor growth (based on volume) (**Figure 5B**). The dose 5 mg/kg was the most effective concentration of rU1 snRNPA in this study. Plasma concentration was monitored and measured by ELISA (**Figure S6A**). The peak plasma concentrations of rU1 snRNPA occurred at 8 h. The absorption and distribution time of rU1 snRNPA *in vivo* were similar to those of durvalumab, but the excretion time was much shorter, and it could hardly be detected in blood after 24 h. This was possibly due to the smaller molecular weight of rU1 snRNPA compared with durvalumab. The organ weight and H&E staining results showed that the effects of rU1 snRNPA treatment on important organs (heart, liver, spleen, lung, and kidney) were similar with those of durvalumab, with no increase in damage to the histology of these organs (**Supplementary Figures S6B**, **S7**). Collectively, these results indicated that rU1 snRNPA possessed ideal *in vivo* anti-melanoma efficacy.

### Key Residues on the Interface of U1 snRNPA/PD-L1 Complex

**Figure 6:**
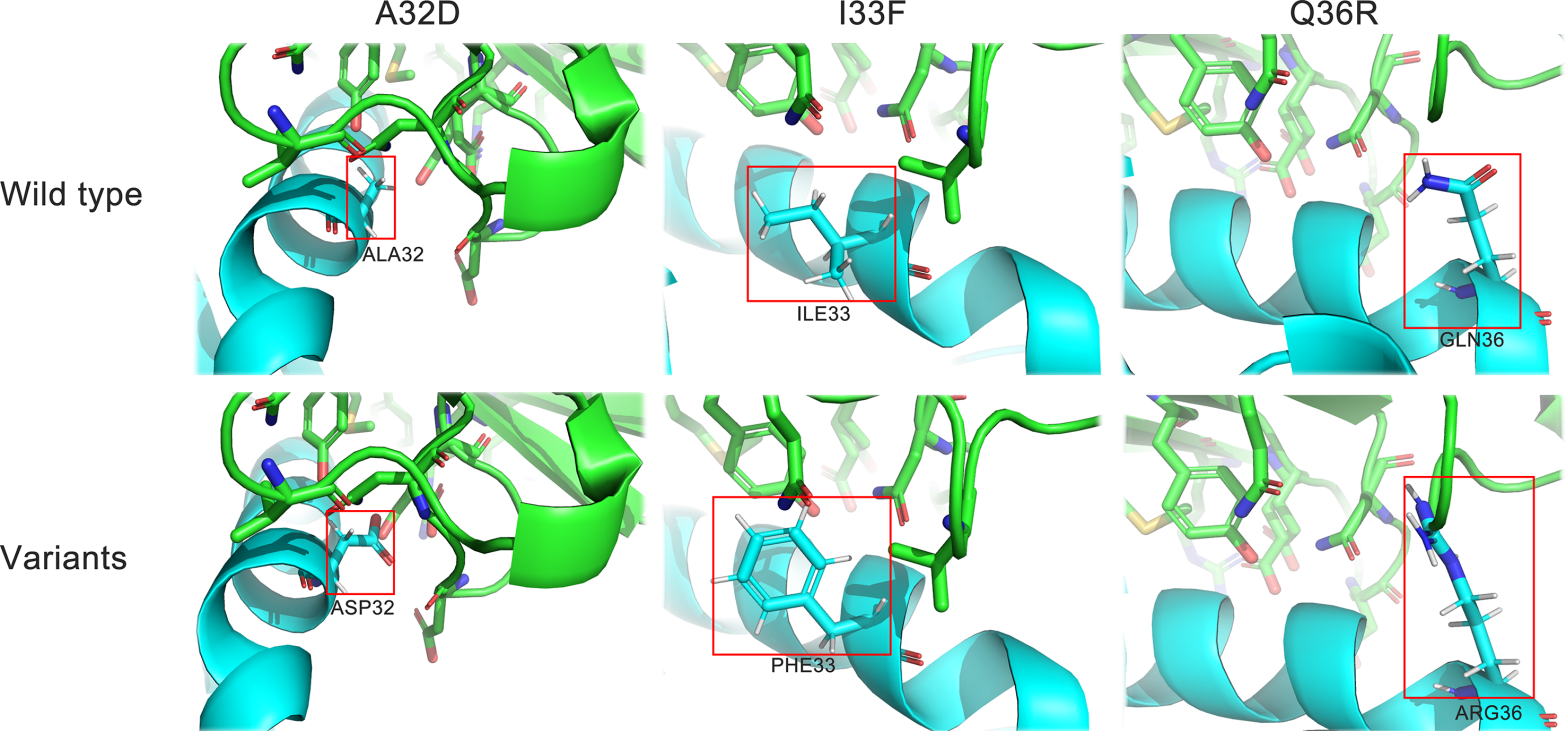
Structure details of the variants. Cyan, U1 snRNPA; green, PD-L1. Stick representations in the red box show the mutated residues.

To further verify that U1 snRNPA worked by binding to PD-L1 and find the key residues for binding, we designed three single-point variants based on the structure of the U1 snRNPA/PD-L1 complex (**Figure 6**). According to the hydrophobic and electrostatic interaction, several potential residues were selected and mutated by Pymol; then, the complexes of variants and PD-L1 were scored by PatchDock. Unlike the previous docking in screening, in this docking, there were certain restrictions on the binding position, and full-length U1 snRNPA was used, so the scores were a little different to the scores of screening. Compared with the scores of wild types, we finally designed the variants of A32D, I33F, and Q36R. Among them, A32D raised the score; others lowered the scores in PatchDock (**Supplementary Table S2**).

**Figure 7:**
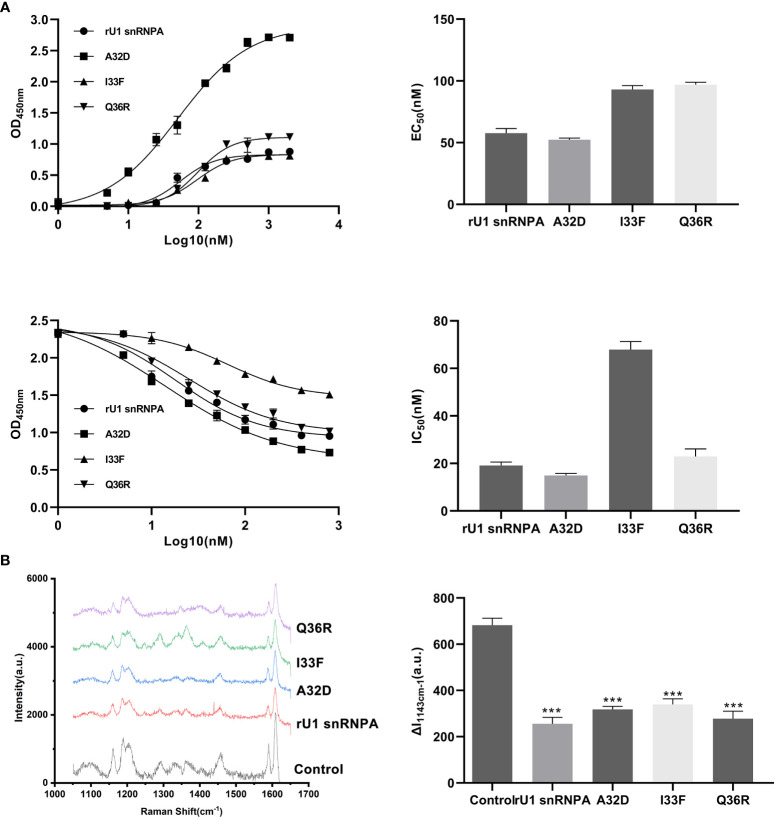
Results of binding ability changes of variants. **(A)** Binding curves and inhibition curves of the variants. Charts of EC50 and IC50. Curve fitting was performed by using GraphPad Prism 8.0. The experiment was performed in double for each sample group. Data are shown as mean ± SD. **(B)** Comparison of the SERS spectra of the variants (both 1.5 μM). The characteristic peaks of 4-ABP are marked in gray. λex = 632.8 nm, t = 5 s, and accumulation times = 1. Data are shown as mean ± SD. ***P < 0.001.

Three variants were expressed and purified in the same way as the wild types and were used for subsequent experiments (**Supplementary Figure S2**). The binding ability to PD-L1 and the PD-1/PD-L1 inhibition efficacy of the variants were evaluated by the same methods as those of the wild types. The results of SERS showed that all variants lowered the characteristic peaks of 4-ABP compared to the control group, but the degree of reductions had no significant difference between the variants, suggesting that SERS may not be suitable for detecting small changes to the PD-1/PD-L1 interaction (**Figure 7B**). The results of ELISA proved that the variants had changed binding ability or inhibition efficacy, compared to wild types, corresponding to theoretical predictions (**Figure 7A**). Among the variants, the EC50 of A32D (51.36 nM) was lower than that in the wild types, which implied that it had better binding ability to PD-L1. The EC50 of others were higher than those in the wild types, which implied that they had worse binding ability to PD-L1. In the results of competitive ELISA, A32D (14.28 nM) had lower IC50, while the others had higher IC50. As the lower IC50 had better inhibitory effects to PD-1/PD-L1 interaction, A32D had better inhibition efficacy than the wild types. Moreover, the results showed that the EC50 and IC50 were not corresponding completely, suggesting that better binding ability to PD-L1 did not mean better inhibition efficacy to the PD-1/PD-L1 interaction. This feature should be emphasized in follow-up research and development. The results mentioned above verified the key residues of U1 snRNPA for binding to PD-L1 and inhibiting the PD-1/PD-L1 interaction. The study of key residues on the interface of the U1 snRNPA/PD-L1 complex was not only to optimize and design the binder but also was conducive to analyze the key residues for interaction theoretically.

### Increased Efficient Therapeutic Variant A32D

**Figure 8:**
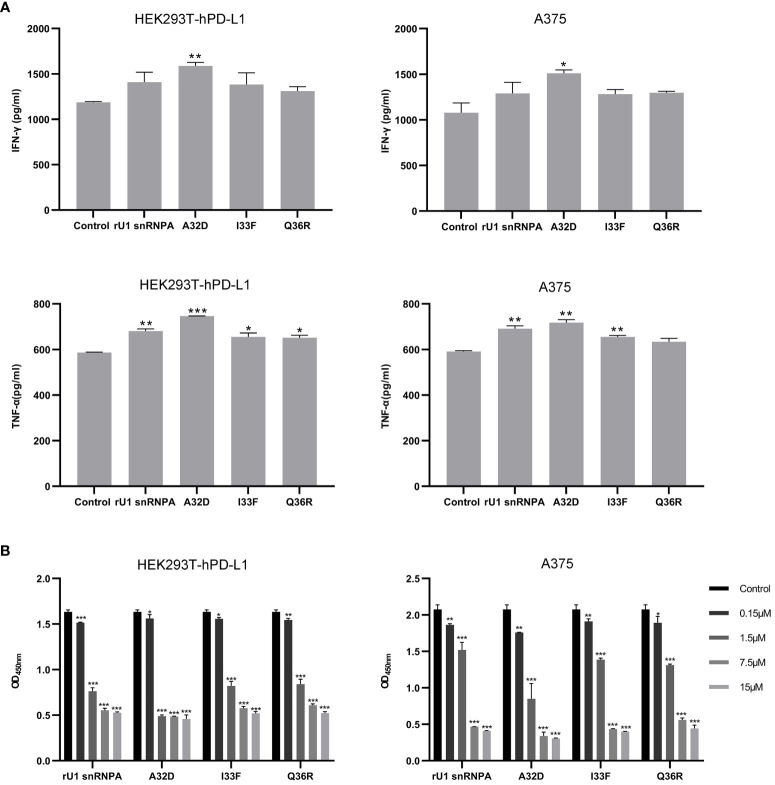
Effect of variants on T cell reactivation function and anti-melanoma efficacy. **(A)** Effect of variants on IFN-γ and TNF-α secretion from CD4+ T cells co-cultured with HEK293T-hPD-L1 cells or A375 cells. **(B)** Anti-melanoma efficacy of variants mixed with CD4+ T cells in HEK293T-hPD-L1 cells or A375 cells. Data are shown as mean ± SD. *P < 0.05, **P < 0.01, ***P < 0.001.

The effect of the variants on bio-activity was evaluated by T cell reactivation function and *in vitro* anti-melanoma efficacy. The experiment methods of the variants were the same as the wild types. In the A32D group of T cell, the reactivation assays showed that the levels of IFN-γ and TNF-α were higher than the wild-type groups, and others were higher than the control group but lower than the wild-type groups, although some groups did not have significant statistical differences (**Figure 8A**). From the results of *in vitro* anti-melanoma assay and killing activity assay, the variants still had efficacy to kill cells which expressed PD-L1 relying on CD4+T cells, and the efficacy was changed compared to the wild types (**Figure 8B**). Generally, *in vitro*, the A32D group had better anti-melanoma efficacy than the other groups, and other variants had similar or lower anti-melanoma efficacy than the wild types. The results of the variants in PD-L1-negative HEK293T cells co-cultured with CD4+ T cells or PD-L1-positive cells not co-cultured with CD4+ T cells did not significantly change the IFN-γ and TNF-α secretion (**Supplementary Figure S8A**) or killing activity (**Supplementary Figure S8B**). These results indicated that the variants of key residues led to the change of T cell reactivation function and *in vitro* anti-melanoma efficacy or killing activity to cells which expressed PD-L1. However, because only one residue had been mutated in each variant, they had not completely lost or most improved their binding ability to PD-L1, so their bio-activities were just partially changed.

## Discussion

In recent years, except for chemical inhibitors, the development of non-IgG PD-L1 inhibitors seems to only focus on PD-1-based variants and peptides (6). So far, there were no other reports about the novel type of PD-L1 inhibitor from human-derived protein scaffolds library. The most important advantage of human-derived protein scaffolds is low or even no immunogenicity. The immunogenicity of therapeutic proteins is one of the serious problems hindering their development, especially when they are administered as multiple doses over prolonged periods (20). There are many factors that can influence the immunogenicity of therapeutic proteins, such as structural features (sequence variation and glycosylation), storage conditions (denaturation or aggregation caused by oxidation), and contaminants or impurities in the preparation (20). The clinical application of antibodies is also challenged by immunogenicity. The immunogenicity of antibody therapeutics can impact the safety and pharmacokinetic properties, which can impact the efficacy of the drugs (21). Therefore, understanding, controlling, and engineering around the potential immunogenicity is of great concern to the pharmaceutical industry (20). Although strategies, such as the humanization of antibodies, have reduced the immunogenicity to a certain extent, there is no solution that can better solve the problem of immunogenicity in the development of protein drugs (21). The human-derived protein scaffolds that we studied provided a new strategy to solve the problem of immunogenicity. Since the scaffolds were originally derived from humans, they have almost no immunogenicity in the human body, avoiding serious side effects in clinical treatment.

In addition, human-derived protein scaffolds are different from PD-L1 antibodies, which can avoid many disadvantages of the antibodies. First, the manufacturing cost of human-derived protein scaffolds like rU1 snRNPA can be lower than that of antibodies which had low yield from mammalian expression systems. Second, the molecular weight of rU1 snRNPA is lower than antibodies, which may lead to better penetration into solid tumors. Compared with PD-1-based variants or peptides, human-derived protein scaffolds like rU1 snRNPA have a more ideal half-life with restriction of molecular weight and are more conducive to becoming an injectable immune-therapy drug. Ideal half-life also makes human-derived protein scaffolds like rU1 snRNPA better to control in terms of appropriate administration dosage to reduce adverse events. Compared with chemical inhibitors, human-derived protein scaffolds can better balance the ideal therapeutic effect and have low cytotoxicity. However, not all human-derived protein scaffolds can be developed into drugs—for example, enzymes and their analogues which interact with some small molecule substrates in the body and play extremely important functions are not suitable for development as therapeutic agents. In the same way, some scaffolds, such as interleukins, which act outside the cell cannot be developed into drugs as well; they are likely to interfere with the normal functions of the human body and cause a series of side effects. The U1 snRNPA selected in this study is located in the nucleus, which acts inside the cell, so it will not interfere with the normal function of the human body when it acts as a therapeutic agent outside the cell. In addition to the type of scaffolds, the molecular weight is also very important. It is necessary to fully consider the filtration of the glomerulus and the reabsorption of the renal tubules (22). In the subsequent development of a novel type of PD-L1 inhibitors from human-derived protein scaffolds library, these factors need to be paid attention to in order to obtain more ideal candidate inhibitors.

In anti-cancer assays, whether in melanoma A375 cells or breast cancer MDA-MB-231 cells or HEK293 cells expressing PD-L1, rU1 snRNPA has almost no anti-cancer activity without tumor-suppressed T cells. These results indicated that the rU1 snRNPA does not work by its own certain anti-tumor activity. Combined with the results of T cell reactivation assays, it can be determined that rU1 snRNPA exerts anti-tumor activity by reactivating tumor-suppressed T cells. Anti-cancer drugs also need to be specific and can target tumor cells but cannot or rarely kill normal cells to avoid serious side effects. In HEK293 cells without PD-L1 expression, rU1 snRNPA, as an inhibitor of PD-L1, had almost no reactivation of tumor-suppressed T cells or killing activity, which can preliminarily prove that rU1 snRNPA had specificity of targeting PD-L1 to exert anti-cancer activity. In the process of new drug development, many candidates have obtained ideal results in *in vitro* assays, but not in *in vivo* assays. Compared with antibodies, the human-derived protein scaffolds that we selected do not have IgG fragments and do not cause antibody-dependent cell-mediated cytotoxicity, so they cannot mediate killer cells to directly kill tumor cells (23). In order to investigate whether rU1 snRNPA can also obtain the ideal anti-cancer activity *in vivo*, we designed a melanoma–human immune system immunodeficiency mouse model to further evaluate the *in vivo* anti-melanoma efficacy of rU1 snRNPA. This mouse model simulated, as much as possible, the anti-tumor activity which rU1 snRNPA may exert on A375 cells in the human immune system. Fortunately, rU1 snRNPA had also achieved ideal results in an *in vivo* anti-melanoma assay, which means that rU1 snRNPA has potential for follow-up research.

## Conclusion

The systematic screening of therapeutic proteins against the drug-target protein is a substantial challenge to *in silico* methods. In this study, the application of a human-derived protein scaffolds library and rigid molecular docking is a highly efficient and easily available tool for designing novel therapeutic protein drugs. Two major biological criteria for the screening of candidates are as follows: (a) do not interact with proteins in body liquids and cell surface receptors, such as cytokines, *etc.*, and (b) do not interact with small molecules related to life activities, such as enzymes. Our work suggested that the variant A32D of rU1 snRNPA, which we screened and improved, is a potential PD-L1 inhibitor.

## Data Availability Statement

The original contributions presented in the study are included in the article/**Supplementary Material**, further inquiries can be directed to the corresponding authors.

## Ethics Statement

The animal study was reviewed and approved by the Institutional Animal Ethics Committee and Animal Care Guidelines of Jilin University.

## Author Contributions

CM and CL conceived the whole project. CL and FS managed the research group. CM and SQ calculated the *in silico* screening. CM and LS conducted the expression and purification. CM measured the bio-activity *in vitro* and SERS detection. ZL and SQ measured the anti-tumor efficacy *in vivo*. All authors contributed to the article and approved the submitted version.

## Funding

This work was supported by the Department of Science and Technology of Jilin Province under grant no. 20190304035YY.

## Conflict of Interest

The authors declare that the research was conducted in the absence of any commercial or financial relationships that could be construed as a potential conflict of interest.

## Publisher’s Note

All claims expressed in this article are solely those of the authors and do not necessarily represent those of their affiliated organizations, or those of the publisher, the editors and the reviewers. Any product that may be evaluated in this article, or claim that may be made by its manufacturer, is not guaranteed or endorsed by the publisher.
