# Supplementary material for: A Novel Type of PD-L1 Inhibitor rU1 snRNPA From Human-Derived Protein Scaffolds Library
Source: Front Oncol. 2021 Nov 29;11:781046. doi: 10.3389/fonc.2021.781046 (PMC8666589; doi:10.3389/fonc.2021.781046)
Supplement: Supplementary file 1 [file DataSheet_1.docx]

Supplementary Material

## Supplementary Materials and Methods

**Protein Expression and Purification**

The coding sequence of full length U1 small nuclear ribonucleoprotein A with a MAP hexa-histidine tag (U1 snRNPA) was ligated into a pSyno-1 vector (Convenience Biology) by seamless cloning and identified by sequence alignment and restriction digests (MluI/XhoI) (Figure S1A). Variants were exactly the same as wild types except for the mutation sites. Variants’ sequences and expression vectors were also synthesized and constructed by Convenience Biology, and identified by sequence alignment and restriction digests.

BL21 (DE3) competent cells were selected as the expression system. The vectors were introduced into BL21 (DE3) competent cells, clones were cultured in LB medium containing kanamycin at 37 °C overnight, and then 5 ml of the culture was transferred to 500 ml LB medium containing 30 μg/ml kanamycin and shaken at 37 °C until the optical density was approximately 0.6. IPTG was added to a final concentration of 0.1 mM was added, followed by shaking at 37 °C for 3 h. Bacteria were collected by centrifugation at 5000 rpm for ultrasonification. The ultrasonification conditions were as follows: 300 W for 2 h on ice (each treatment lasted for 4 s with an interval of 6 s) and centrifugation at 12,000 rpm. The supernatant was added to Ni-NTA and eluted with 50, 100, 200, and 400 mM of imidazole. The eluate at the elution peak was dialyzed in 0.1 M PB (pH=7.8) buffer overnight. The purity of the His-tagged rU1 snRNPA or variants was determined by sodium dodecyl sulfate polyacrylamide gel electrophoresis (SDS-PAGE) (Figure S2). Western blotting with horseradish peroxidase (HRP) Anti-6×His tag® antibody was used to identify the proteins (Figure S3).

**Establishment of SERS-based PD-1/PD-L1 inhibitor detection platform and experimental process**

Recently, Surface Enhanced Raman Scattering (SERS) as a fingerprint analysis technology has received more and more attention. It has significant advantages such as non-destructive data collection, single-molecule sensitivity, fast response time and unique information. In the fields of bio-medicine, SERS has potential applications, such as the detection of tumor markers. However, there is no report on the use of SERS for the detection of PD-1/PD-L1 interactions.

In this study we designed a SERS-based PD-1/PD-L1 inhibitor detection platform (Scheme S1) and the feasibility of this platform was verified by Durvalumab and BMS-202(Figure S4C and D). Durvalumab is an IgG1 monoclonal antibody that has high affinity binding to PD-L1 receptor and BMS-202 is a non-peptide small molecule inhibitor that Bristol-Myers Squibb Company (BMS) recently disclosed for the PD-1/PD-L1 pathway. They respectively represent antibody inhibitors and small molecule non-peptide inhibitors that have been confirmed to inhibit the PD-1/PD-L1 signaling pathway, so they are used to verify the feasibility of the method.

The AgNPs (Ag NanoParticles) used in the platform were synthesized by the citrate reduction reaction according to Lee’s method. The achieved AgNPs have a plasmonic band located at 428 nm (Figure S4A), and the transmission electron microscopic (TEM) image shows they are all in a quasi-spherical shape with an average size of 36 nm (Figure S4B).To prevent the aggregation of AgNPs during surface modification, 1.0 ml of AgNPs (0.30nM) and 10 μl of Tween 20 (74 μM) were gently mixed for 30 min. The carboxylate terminal groups on the surfaces of AgNPs were activated by adding 5.0μl of N-ethyl-N0-(3-(dimethylamino)propyl) carbodiimide (EDC) and N-hydrox-ysuccinimide (NHS) (both 2.5mM) for subsequent chemical bonding to antibodies. After 1 h activation, the unreacted molecules were removed by centrifugation at 4000g for 7 min. Next, 3.0 μl of 1 mg/mL PD-1 (Genscript, Z03370) and 20μl of SERS reporter 4-ABP (5.0 mM) were mixed with the AgNPs solution (1.0 ml, 0.30nM) for 2 h at room temperature, and unreacted molecules were removed by centrifugation (4000g for 7 min).Nonspecific binding chemicals and antibodies were washed by centrifugation, and the remaining antibody-conjugated AgNPs were resuspended in the PBS buffer solution. Thus, the prepared AgNPs@PD-1@4-ABP was ready for use. Next, we conjugated PD-L1 (Genscript, Z03371) onto the surfaces of magnetic beads. First, 400 μl of 0.5 mg/ml carboxylic group-functionalized magnetic beads was activated by 5.0 μl of 100mM EDC and NHS dissolved in distilled water for 1 h. Then, the magnetic beads were separated using a magnet and washed with PBS buffer solution to remove unreacted molecules. After the magnetic beads were resuspended with PBS buffer, 3.0 μl of PD-L1 (1 mg/mL) were added to the magnetic bead solution and reacted for 2 h under stirring at room temperature. Magnetic beads were washed three times to remove nonspecifically bound PD-L1, followed by resuspending in the PBS buffer solution to achieve MNs@PD-L1. After the SERS probes were synthesized successfully according to the above methods, they were configured as a detection platform according to the ratio of 3:1 (AgNPs@PD-1@4-ABP:MNs@PD-L1), and a certain volume of additions were added to the system respectively, and the control group add an equal volume of PBS or DMSO. The characteristic peaks of 4-ABP at 1143cm^-1^ and 1600cm^-1^were used as the observation index to judge whether the peak height changes, then to determine whether the additions had inhibition effect on PD-1/PD-L1 interaction.

**Binding ELISA and competitive ELISA**

Human PD-L1 (hPD-L1) was purchased from SinoBiological. 96-well plates were precoated with 1 µg/ml hPD-L1 at 4 °C overnight. Then, the plates were washed with phosphate-buffered saline with Tween 20 (pH 7.4) three times and blocked with 5% bovine serum albumin for 2 h. Afterwards, different concentrations of rU1 snRNPA, Durvalumab or variants were added and incubated for 2 h at 37 °C.

For PD-1 competitive ELISA, rU1 snRNPA, Durvalumab or variants were co-incubated with 2 µg/ml human PD-1 (Genscript®) for 30 min at room temperature and then transferred to 96-well plates precoated with hPD-L1 (1 µg/ml), followed by incubation for 2 h at 37 °C.

For binding ELISA, an HRP Anti-6×His tag® antibody was added after the final incubation to detect rU1 snRNPA binding according to the manufacturer’s instructions. For PD-1 competitive ELISA, PD-1 antibody (Abcam) and goat anti-mouse IgG H&L (HRP) antibody (Abcam) were added after the final incubation to detect PD-1 binding according to the manufacturer’s instructions.

Binding affinity was determined by absorbance at 450 nm wavelength through a microplate reader (Tecan Austria GmbH 5082 Grodig).Half-maximal effective concentration (EC_50_) values and half-maximal inhibitory concentration (IC_50_) values were calculated using GraphPad Prism version 8.0 to evaluate the binding ability or competitive potency of rU1 snRNPA.

**Construction of PD-L1 expression cell line**

Human embryonic kidney cells (HEK293T, Procell),human melanoma cells (A375, Procell) and human breast adenocarcinoma cells (MDA-MB-231,Procell) were cultured in DMEM medium supplemented with 10% (v/v) FBS and 100 units/ml penicillin–streptomycin (Gibco Invitrogen).

The hPD-L1 DNA sequence was synthesized by Convenience Biology and ligated to the mApple-N1 plasmid (supplied by the Institute of Biophysics, Chinese Academy of Sciences, Beijing, China) by the XhoI and XbaI restriction sites (Figure S1B),and then transfected into HEK293T cells using Invitrogen Lipofectamine™ 3000 transfection reagent according to the manufacturer’s instructions.

rU1 snRNPA and Durvalumab were conjugated with Alexa Fluor® 488 NHS Ester (Invitrogen) and purified by molecular sieving to obtain conjugates. A375 cells with high expression levels of PD-L1 were incubated with different concentrations of rU1 snRNPA or Durvalumab. The conjugates and cells were observed with the high-content Operetta CLS imaging system (PerkinElmer).

***In vitro* anti-cancer assay**

*In vitro* anti-cancer efficacy of rU1 snRNPA and variants was determined by Cell Counting Kit 8 (CCK8) (Beyotime) assay. According to the manufacturer’s instructions, 20,000 A375 cells, MDA-MB-231 cells(not in variants’ experiments),HEK293T-hPD-L1 cells or PD-L1-negative HEK293T cells were inoculated into each well of 96-well plates respectively and adhered for 24h.Then the cells cultured in 100 μl medium containing 2% fetal bovine serum (FBS) with different concentrations of rU1 snRNPA or variants with or without mixing with CD4^+^ T cells for 48 h.

10 μl CCK-8 was added to the wells, followed by incubation for 2 h. Optical density values at 450 nm wavelength were measured with a microplate reader (Tecan Austria GmbH 5082 Grodig).

***In vivo* anti-melanoma assay**

NOD/SCID mice were inoculated subcutaneously with human melanoma cells A375 (5 × 10^6^) mixed with human PBMCs (1 × 10^6^) on the middle abdomen. The mice were divided into rU1 snRNPA group and Durvalumab group, each of which contained three subgroups (1mg/kg, 5mg/kg, and 10mg/kg). Blank control and negative control were also established. Each group contained 10 mice. The mice were treated intraperitoneally twice per week from the day of inoculation. Tumor volumes were monitored twice a week as follows: three orthogonal axes (a, b, and c) were measured, and the tumor volume was calculated as (a × b × c) / 2.

*In vivo* pharmacokinetics of rU1 snRNPA was detected as follows: The mice were divided into two main groups (rU1 snRNPA and Durvalumab) and treated intraperitoneally with 5mg/kg. Blood samples were taken at 0 min, 15 min, 30 min, 45 min, 1 h, 2 h, 3 h, 4 h, 6 h, 8 h, 10 h, 12 h, 24 h, 48 h, and 72 h after injection, and serum was collected for testing. The concentrations of rU1 snRNPA *in vivo* were monitored by ELISA (Figure S7A).

The weights of heart, liver, spleen, lung, and kidney were measured (Figure S7B). All institutional and national guidelines for the care and use of laboratory animals were followed. All animal experiments were conducted in accordance with the Institutional Animal Ethics Committee and Animal Care Guidelines of Jilin University and approved by the hospital ethics committee (Approval No. 20200051).

Histological changes in heart, liver, spleen, lung, and kidney tissues were observed using H&E staining to further assess the potential adverse effects associated with rU1 snRNPA. For perfusion fixation, tissues were placed in 4% formaldehyde, dehydrated using an ethanol gradient, and vitrified in dimethylbenzene. Samples were embedded in paraffin, stained with H&E, and observed under a microscope (DS-Fi1, Nikon) at 100× magnification (Figure S8).

## Supplementary Figures


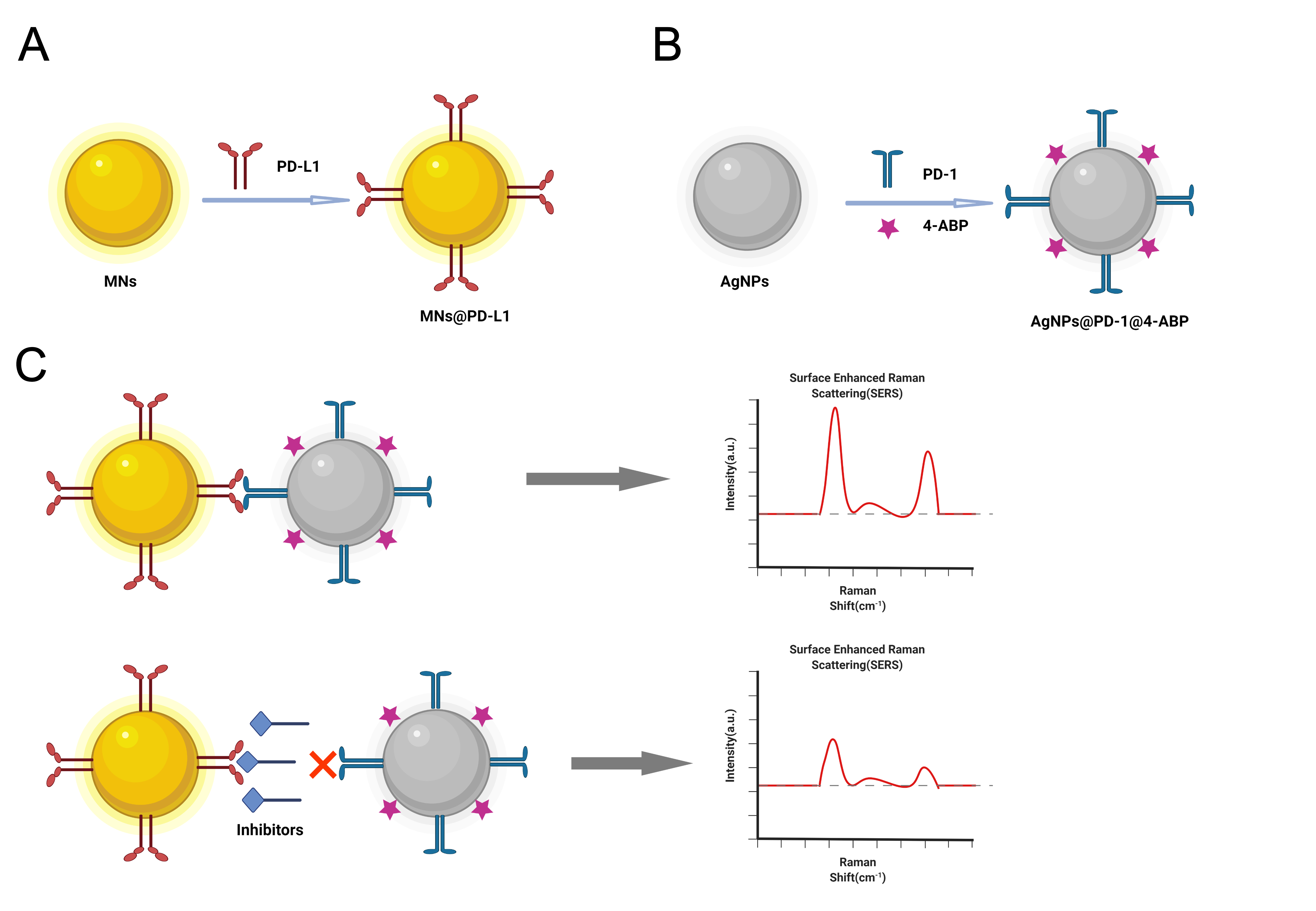


**Supplementary Scheme S1.**(A)Schematic illustration of the fabrication processes of PD-L1-conjugated MNs.(B)Schematic illustration of the fabrication processes of PD-1-conjugated AgNPs modified with Raman reporter 4-ABP.(C)The interpretation of the mechanism of SERS-based PD-1/PD-L1 inhibitor detection platform. The scheme was created with BioRender.com.


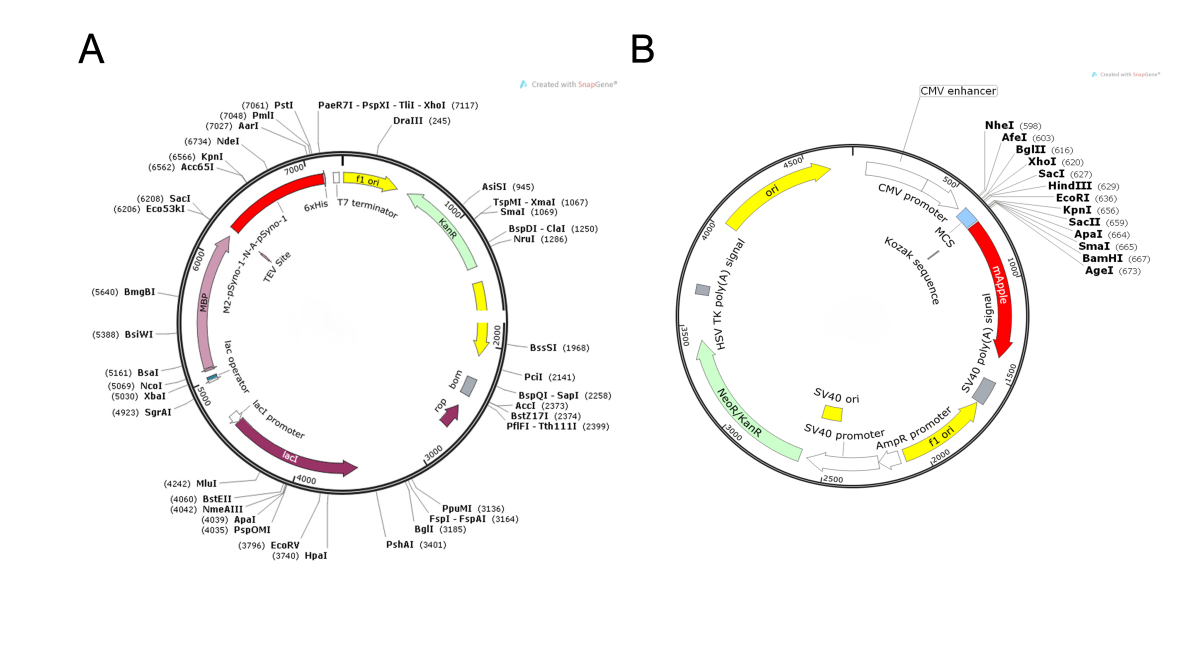


**Supplementary Figure S1.**Plasmid maps in this study. (A)Full length U1 snRNPA with a MAP hexa-histidine tag was cloned into pSyno-I vector. After construction of the wild type plasmids, PCR-based mutagenesis was performed to generate the mutants of rU1 snRNPA.(B)Plasmid map of mApple-N1.mApple is a basic red fluorescent protein which can express red fluorescence in cells .


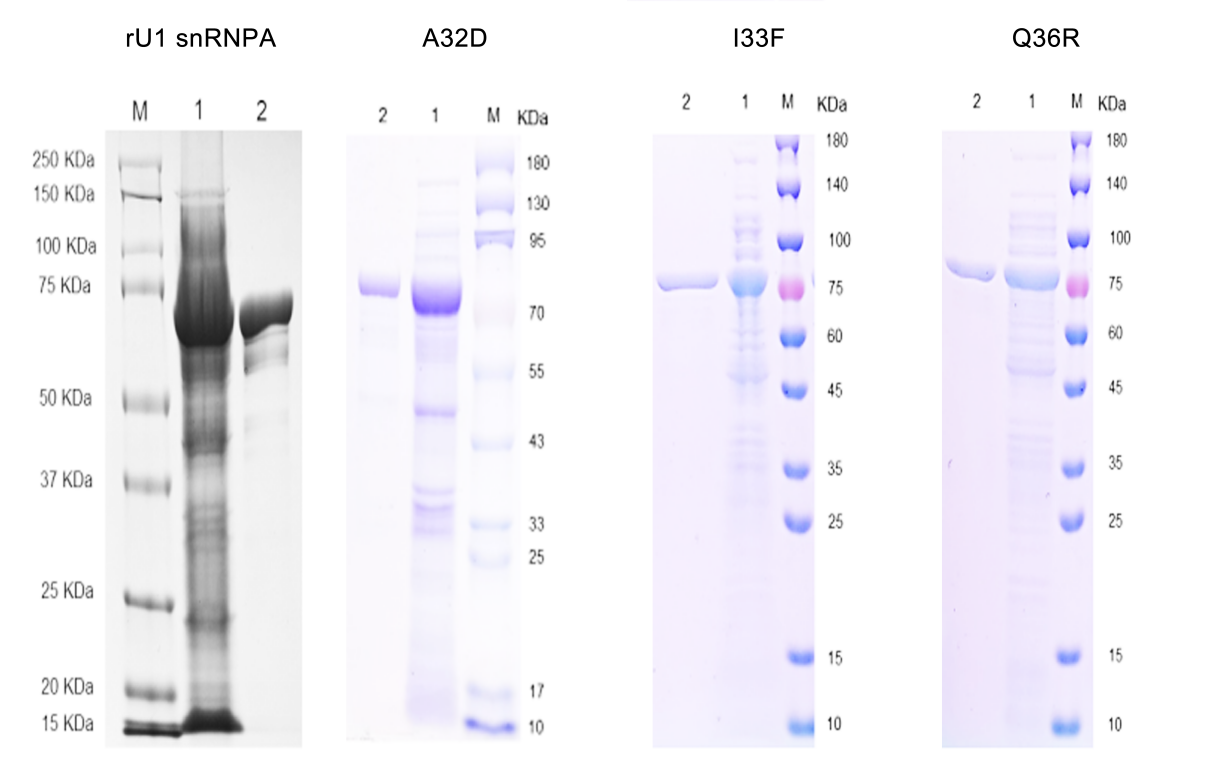


**Supplementary Figure S2.**SDS-PAGE results during rU1 snRNPA or variants production and purification. Lane 1:rU1 snRNPA or variants before purification, Lane 2:rU1 snRNPA or variants after purification.


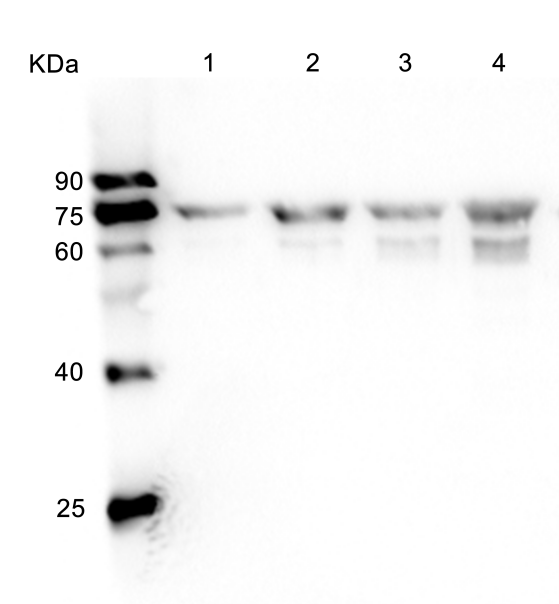


**Supplementary Figure S3.**WB results of rU1 snRNPA and variants by Anti-6×His tag antibody. Lane 1-4: Q36R, I33F, A32D, rU1 snRNPA


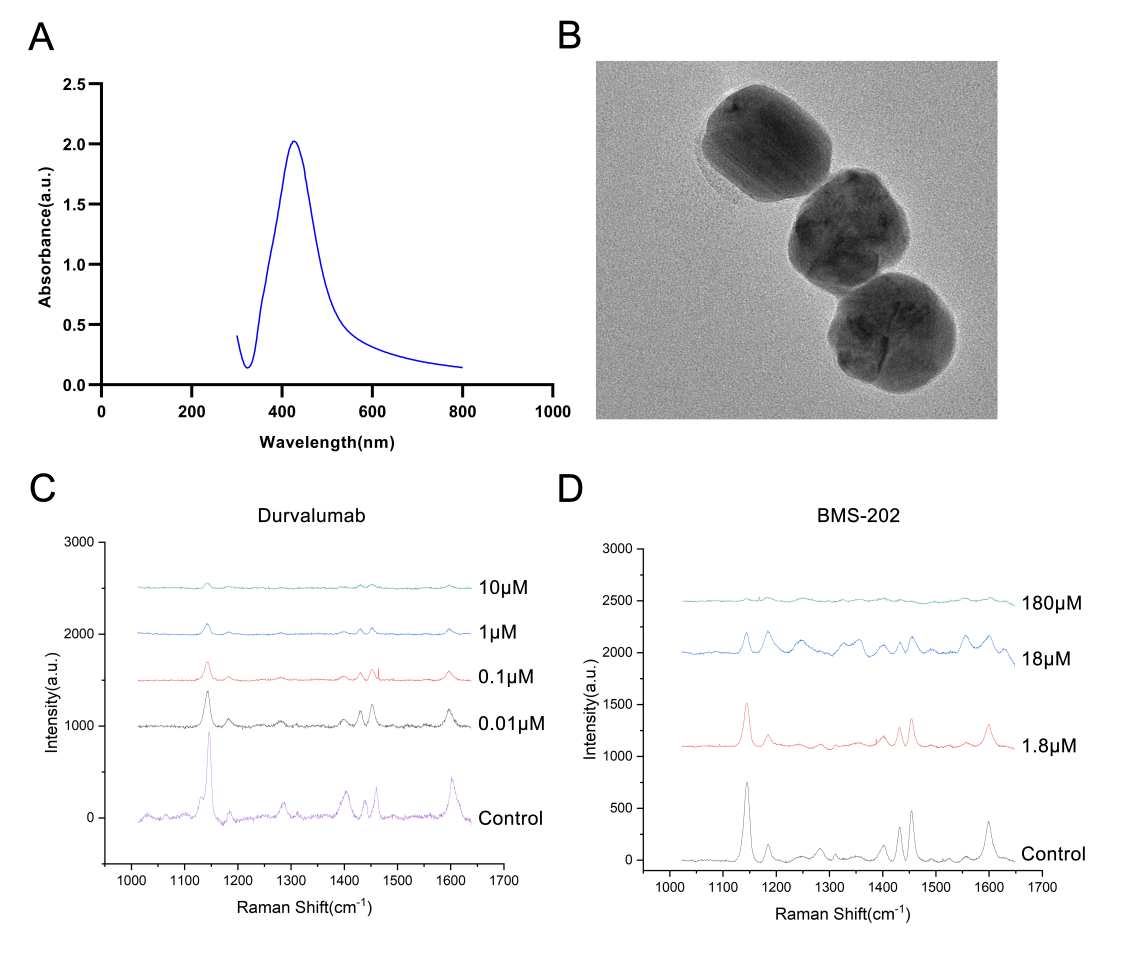


**Supplementary Figure S4.**Results of establishment of SERS-based PD-1/PD-L1 inhibitor detection platform. (A)UV-vis spectra of AgNPs. (B)TEM images of AgNPs. (C)Concentration-dependent SERS spectra of Durvalumab. (D)Concentration-dependent SERS spectra of BMS-202.





**Supplementary Figure S5.** (A)Effect of rU1 snRNPA or Durvalumab on IFN-γ and TNF-α secretion from CD4^+^ T cells co-cultured with PD-L1 negative HEK293Tcells.(B)Anti-cancer efficacy of rU1 snRNPA or Durvalumab mixed without CD4+ T cells in HEK293T-hPD-L1 cells or A375 cells or MDA-MB-231 cells or PD-L1-negative HEK293T cells with CD4+ T cells. Data are shown as mean±SD. No significant difference from the control group.





**Supplementary Figure S6.** (A)Concentration changes in the blood of mice of rU1 snRNPA or Durvalumab. (B)Weight of heart, liver, spleen, lung, kidney.


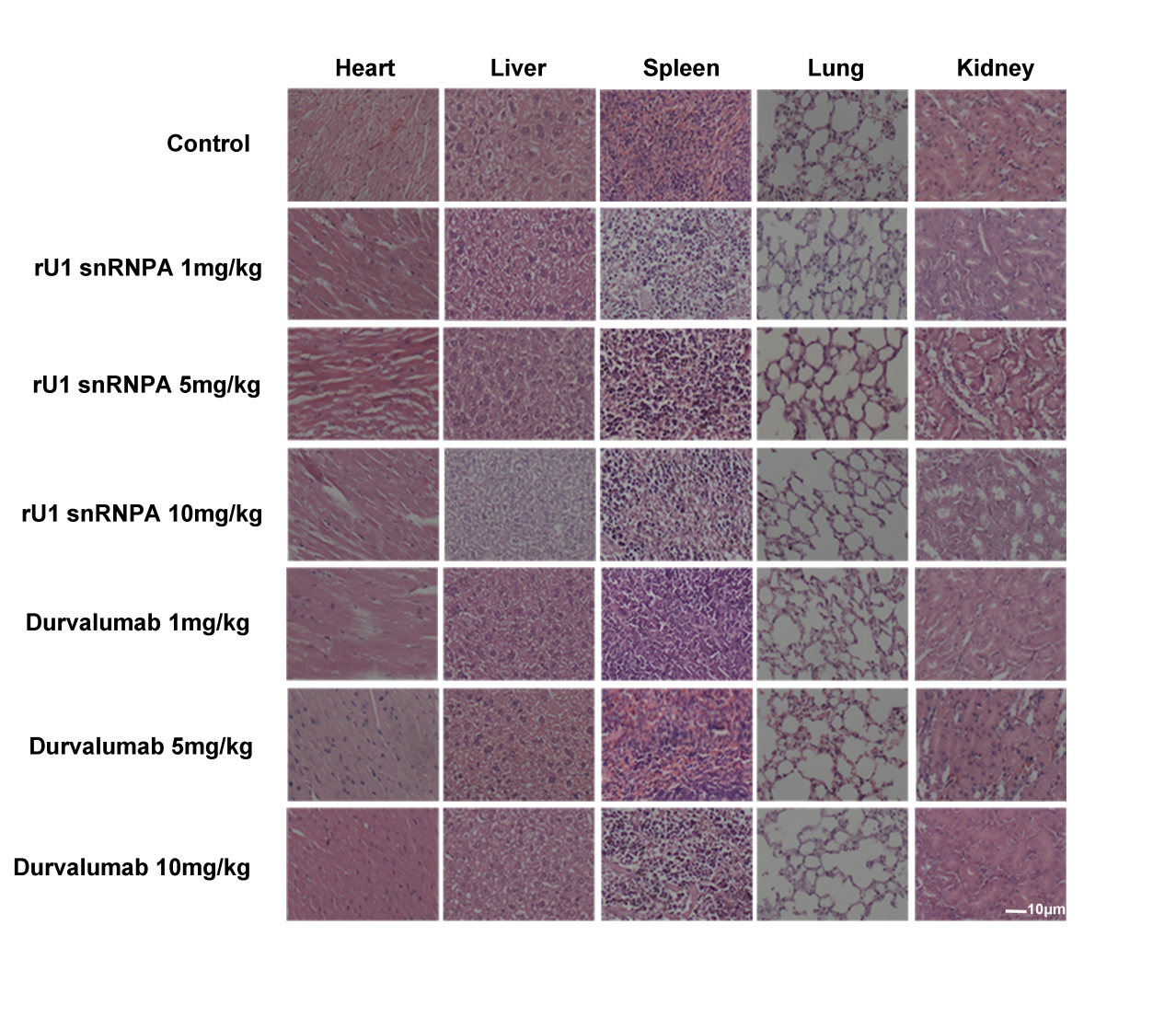


**Supplementary Figure S7.**Histological features of different organs were detected by H&E staining. Scale bar, 10 μm.


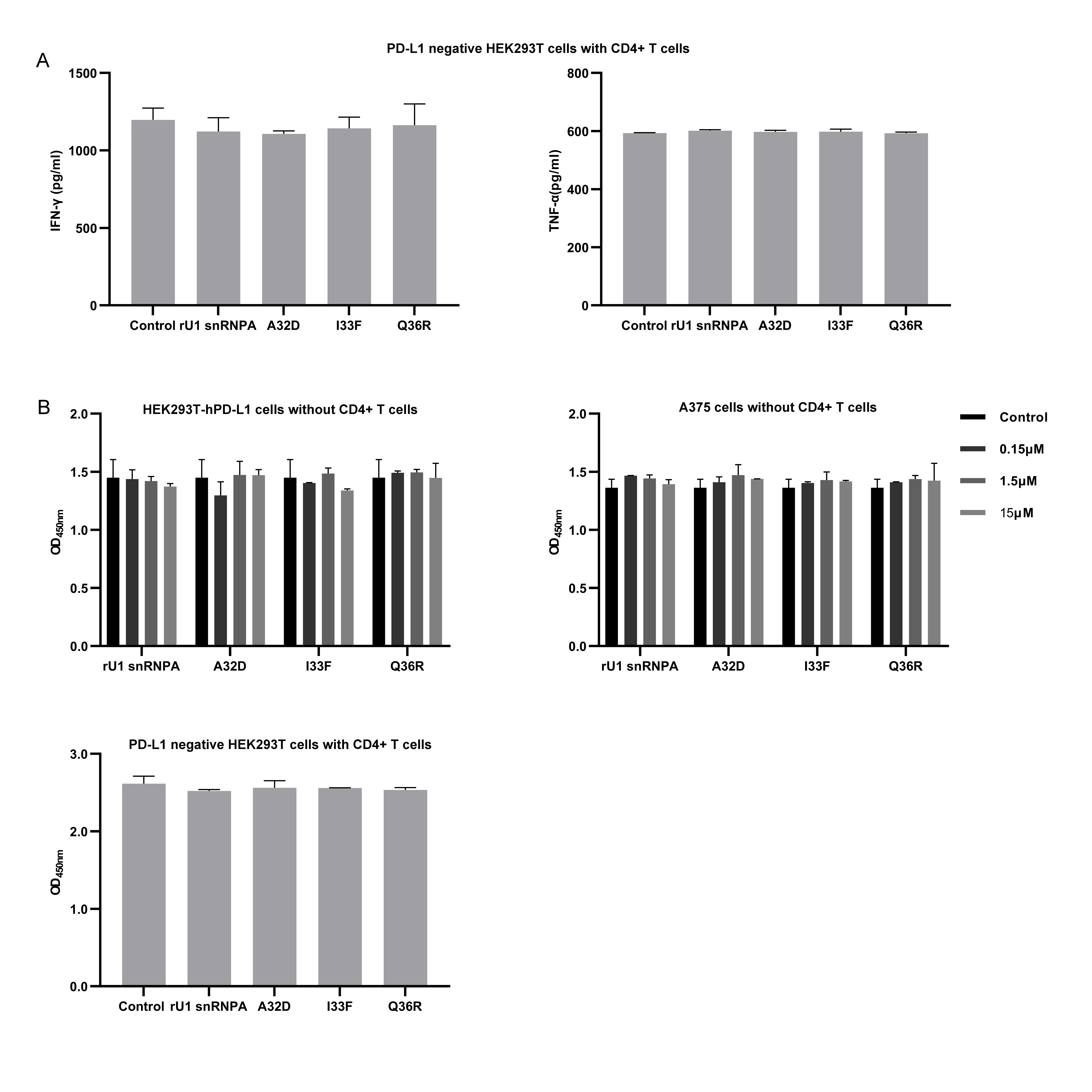


**Supplementary Figure S8.** (A)Effect of variants on IFN-γ and TNF-α secretion from CD4^+^ T cells co-cultured with PD-L1 negative HEK293T cells. (B)Anti-melanoma efficacy of variants mixed without CD4+ T cells in HEK293T-hPD-L1 cells or A375 cells or PD-L1-negative HEK293T cells with CD4+ T cells .Data are shown as mean±SD. No significant difference from the control group.

**3.Supplementary** **Tables**

**TableS1 The top 20 binders screened for the third round**

|  | Name | Score | Classification | Subcellular localization | Physiological effect | Expression System |
| --- | --- | --- | --- | --- | --- | --- |
| 1 | CNTN5 FN1-FN3 domains | 18436 | CELL ADHESION | Cell membrane/GPI-anchor | Contactins mediate cell surface interactions during nervous system development. | Escherichia coli BL21(DE3) |
| 2 | Cytochrome P450 3A4 | 18350 | OXIDOREDUCTASE | Endoplasmic reticulum | A cytochrome P450 monooxygenase involved in the metabolism of sterols, steroid hormones, retinoids and fatty acids | Escherichia coli |
| 3 | Macrophage capping protein | 18130 | STRUCTURAL PROTEIN | Nucleus | Calcium-sensitive protein which reversibly blocks the barbed ends of actin filaments but does not sever preformed actin filaments. May play an important role in macrophage function. May play a role in regulating cytoplasmic and/or nuclear structures through potential interactions with actin. May bind DNA. | Escherichia coli BL21(DE3) |
| 4 | U1 small nuclear ribonucleoprotein A | 17660 | STRUCTURAL PROTEIN | Nucleus | Component of the spliceosomal U1 snRNP, which is essential for recognition of the pre-mRNA 5' splice-site and the subsequent assembly of the spliceosome. | Escherichia coli |
| 5 | Interleukin-1beta converting enzyme | 17082 | HYDROLASE | Cell membrane/Cytoplasm | Interleukin-1beta converting enzyme (ICE/caspase-1) is the protease responsible for interleukin-1beta (IL-1beta) production in monocytes. | Escherichia coli |
| 6 | Glutathione reductase | 17066 | GLUTATHIONE REDUCTASE | cytosol | Exhibits glutathione-dependent thiol transferase and dehydroascorbate reductase activities. | Escherichia coli |
| 7 | HLA class I histocompatibility antigen, B alpha chain,B*0801 | 17064 | IMMUNE SYSTEM | Cell membrane/Single-pass type I membrane protein /Endoplasmic reticulum membrane | Antigen-presenting major histocompatibility complex class I (MHCI) molecule.Presents to CD8-positive T cells viral epitopes derived from EBV/HHV-4 EBNA3 (QAKWRLQTL), eliciting cytotoxic T cell response. | Escherichia coli |
| 8 | HLA class I histocompatibility antigen, B-7 alpha chain | 16976 | IMMUNE SYSTEM | Cell membrane/Single-pass type I membrane protein /Endoplasmic reticulum membrane | Displays peptides sharing a common signature motif | Escherichia coli BL21(DE3) |
| 9 | HLA class I histocompatibility antigen, B-35 alpha chain | 16906 | IMMUNE SYSTEM | Cell membrane/Single-pass type I membrane protein /Endoplasmic reticulum membrane | Antigen-presenting major histocompatibility complex class I (MHCI) molecule. | Escherichia coli BL21(DE3) |
| 10 | Poly [ADP-ribose] polymerase 1/human PARP1 catalytic domain | 16888 | TRANSFERASE | Nucleus/nucleolus/Chromosome | Poly-ADP-ribosyltransferase that mediates poly-ADP-ribosylation of proteins and plays a key role in DNA repair | Escherichia coli |
| 11 | Methionine aminopeptidase 2 | 16596 | HYDROLASE | Cytoplasm（About 30% of expressed METAP2 associates with polysomes.） | otranslationally removes the N-terminal methionine from nascent proteins. | Escherichia coli |
| 12 | PC4 and SFRS1-interacting protein,Protein IWS1 homolog | 16550 | TRANSCRIPTION | Nucleus | Transcription factor which plays a key role in defining the composition of the RNA polymerase II (RNAPII) elongation complex and in modulating the production of mature mRNA transcripts. | Escherichia coli |
| 13 | DNA polymerase eta | 16450 | Transferase | Nucleus/Chromosome | DNA polymerase that promotes microhomology-mediated end-joining (MMEJ), an alternative non-homologous end-joining (NHEJ) machinery triggered in response to double-strand breaks in DNA | Escherichia coli |
| 14 | Histone-binding protein RBBP4 | 16388 | TRANSCRIPTION | Nucleus | Core histone-binding subunit that may target chromatin assembly factors, chromatin remodeling factors and histone deacetylases to their histone substrates in a manner that is regulated by nucleosomal DNA. | Homo sapiens |
| 15 | DNA polymerase beta | 16286 | REPLICATION | Nucleus | Repair polymerase that plays a key role in base-excision repair. Has 5'-deoxyribose-5-phosphate lyase (dRP lyase) activity that removes the 5' sugar phosphate and also acts as a DNA polymerase that adds one nucleotide to the 3' end of the arising single-nucleotide gap. | Escherichia coli |
| 16 | HLA class I histocompatibility antigen, B-15 alpha chain | 16212 | IMMUNE SYSTEM | Cell membrane/Single-pass type I membrane protein /Endoplasmic reticulum membrane | Antigen-presenting major histocompatibility complex class I (MHCI) molecule. | Escherichia coli |
| 17 | Mitogen-activated protein kinase 10 | 16202 | TRANSFERASE | Mitochondrion /Nucleus/Cytoplasm/Membrane | Serine/threonine-protein kinase involved in various processes such as neuronal proliferation, differentiation, migration and programmed cell death. | Escherichia coli BL21(DE3) |
| 18 | HLA class I histocompatibility antigen,BW-53 B*5301 alpha chain | 16200 | IMMUNE SYSTEM | Cell membrane/Single-pass type I membrane protein /Endoplasmic reticulum membrane | Antigen-presenting major histocompatibility complex class I (MHCI) molecule. | Escherichia coli |
| 19 | Cyclic GMP-AMP synthase | 16182 | TRANSFERASE | Cell membrane/Nucleus/cytosol | Nucleotidyltransferase that catalyzes the formation of cyclic GMP-AMP (cGAMP) from ATP and GTP and plays a key role in innate immunity | Escherichia coli |
| 20 | HLA class I histocompatibility antigen, A-2 alpha chain | 16154 | IMMUNE SYSTEM | Cell membrane/Single-pass type I membrane protein /Endoplasmic reticulum membrane | In complex with B2M/beta 2 microglobulin displays primarily viral and tumor-derived peptides on antigen-presenting cells for recognition by alpha-beta T cell receptor (TCR) on HLA-A-restricted CD8-positive T cells, guiding antigen-specific T cell immune response to eliminate infected or transformed cells | Escherichia coli |

**TableS2 The scores of variants by PatchDock**

| Name | Score |
| --- | --- |
| U1 snRNPA | 9244 |
| A32D | 9444 |
| I33F | 8714 |
| Q36R | 9014 |
